# Dynamic multidrug recognition by multidrug transcriptional repressor LmrR

**DOI:** 10.1038/srep06922

**Published:** 2014-11-18

**Authors:** Koh Takeuchi, Yuji Tokunaga, Misaki Imai, Hideo Takahashi, Ichio Shimada

**Affiliations:** 1 Biomedicinal Information Research Center & Molecular Profiling Research Center for Drug Discovery, National Institute of Advanced Industrial Science and Technology, Aomi 2-3-26, Koto-ku, Tokyo 135-0064, Japan; 2 JST, PRESTO, Aomi 2-3-26, Koto-ku, Tokyo 135-0064, Japan; 3 Research and Development Department, Japan Biological Informatics Consortium, Tokyo, Japan. Aomi 2-3-26, Koto-ku, Tokyo 135-0064, Japan; 4 Graduate School of Medical Life Science, Yokohama City University, Kanagawa 230-0045, Japan; 5 Graduate School of Pharmaceutical Sciences, The University of Tokyo, 7-3-1 Hongo, Bunkyo-ku, Tokyo 113-0033, Japan

## Abstract

LmrR is a multidrug transcriptional repressor that controls the expression of a major multidrug transporter, LmrCD, in *Lactococcus lactis*. However, the molecular mechanism by which LmrR binds to structurally unrelated compounds and is released from the promoter region remains largely unknown. Here, we structurally and dynamically characterized LmrR in the *apo*, compound-bound and promoter-bound states. The compound-binding site of LmrR exhibits ps–μs dynamics in the *apo* state, and compound ligation shifts the preexisting conformational equilibrium to varying extents to achieve multidrug recognition. Meanwhile, the compound binding induces redistribution of ps–ns dynamics to the allosteric sites, which entropically favors the high-affinity recognition. Furthermore, the reciprocal compound/promoter binding by LmrR is achieved by the incompatible conformational ensembles between the compound- and promoter-bound states. Collectively, the data show how LmrR can dynamically exert its functions through promiscuous multi-target interactions, in a manner that cannot be understood by a static structural view.

The acquisition of multidrug resistance (MDR) by pathogenic bacteria is a major threat in the treatment of infectious diseases. Up-regulation of the expression of multidrug transporters that expel toxic compounds from cells is a strategy commonly employed by bacteria with MDR ability^1^,^2^,^3^. The expression of multidrug transporters is strictly regulated by transcriptional activators and/or repressors, which tend to have the ability to bind multiple toxic compounds^4^. In addition, the toxic compounds that are recognized by the multidrug transcriptional regulator and excreted by the transporters are often the same. Thus, drug excretion systems consisting of a set of multidrug transcriptional regulators and transporters efficiently upregulates the expression of the required multidrug transporters in response to toxic compounds. Similar systems are also well-documented in eukaryotes, especially in human cancers^5^,^6^.

A Gram-positive bacterium, *Lactococcus lactis,* exhibits MDR when exposed to increasing concentrations of structurally unrelated toxic compounds, such as Hoechst 33342 (H33342), daunomycin, ethidium, and rhodamine 6G (Rho6G)^7^,^8^ (Fig. 1a). The MDR phenotype is induced by the overexpression of a heterodimeric multidrug transporter, LmrCD^9^. The expression of LmrCD is regulated by a multidrug transcriptional repressor, LmrR, which is encoded in the same gene cluster as the *lmrCD* gene^10^. LmrR is a homodimeric protein that belongs to the PadR-like family of multidrug transcriptional regulators^4^,^11^. In the absence of the compound, LmrR binds to the promoter regions of the *lmrCD* genes to repress their transcription (Fig. 1b). When the cells are exposed to the toxic compounds, LmrR is released from the promoter regions to induce membrane expression of the transporter^12^. LmrR has been shown to bind the aforementioned compounds with relatively high affinities^13^, thus, is a major sensory molecule that controls multidrug resistance in *L. lactis*.

The structures of LmrR in the *apo* state and in complexes with H33342 or daunomycin have been determined^13^. The LmrR dimer contains winged helix–turn–helix (wHTH) DNA-binding motif on the side of the molecule (Fig. 1c), which is thought to fit into successive DNA major grooves with the DNA-recognition helix, α3. The α4 helix, together with the α1 helix, forms a hydrophobic pore at the dimeric center to form the compound binding site. The compound binding has been proposed to change the orientation of the C-terminal α4 helix relative to the wHTH domain (Fig. 1d). In the *apo* and H33342-bound structures, the α4 helix is in an *upper* orientation. In contrast, the α4 helix lies more horizontally and adopts *lower* orientations in the daunomycin-bound structures. Thus, the common structural change upon the compound binding, which leads to the release of LmrR from the promoter regions, has not been identified. Nevertheless, since each of the three X-ray structures of LmrR are derived from different crystal forms, it is not possible to determine whether the different orientations are induced by compound binding or simply reflect differences in crystal packing^13^. The α4-helix orientations are correlated with the relative orientation between the DNA-binding α3 helices of LmrR. The distance between the α3 helices decreases with the *lower* α4-helix orientations than in the *upper* orientations^13^. However, none of the LmrR structures determined thus far is compatible with binding to B-form DNA. Therefore, the structural analysis of LmrR in the promoter-bound state as well as in the compound-bound states in solution, without any distortion caused by experimental conditions, is required, to reveal the molecular mechanism by which LmrR binds to various structurally unrelated compounds and is released from the promoter region to show the MDR phenotype.

## Results

### Dynamics of LmrR in the *apo* state

To characterize the dynamic nature of LmrR, we subjected the protein to solution NMR measurements. The backbone dynamics of *apo* LmrR were analyzed by the longitudinal and transverse ^15^N relaxation rates (R_1_ and R_2_, respectively), as well as the (^1^H)–^15^N heteronuclear nuclear Overhauser effects (NOEs). The mainchain ^15^N relaxation analyses clearly indicated the existence of slow μs-ms chemical exchange at the compound-binding site, which is reflected in the large R_2_/R_1_ values observed for the residues in the α1 and α4 helices (Supplemental Fig.1a). Since the ^15^N R_2_ was insensitive in Carr-Purcell-Meiboom-Gill (CPMG) experiments up to 1 kHz, the time scale of the dynamics in compound-binding site is much faster than the ms range. In addition, the low (^1^H)–^15^N heteronuclear NOE values indicated that part of the α4 helix exhibits substantial rapid internal dynamics on a ps–ns timescale (Supplemental Fig. 1b). Taken together, the compound-binding site of LmrR, especially the C-terminal α4 helix, displays intensive ps–μs dynamics in the *apo* state.

**Figure 1:**
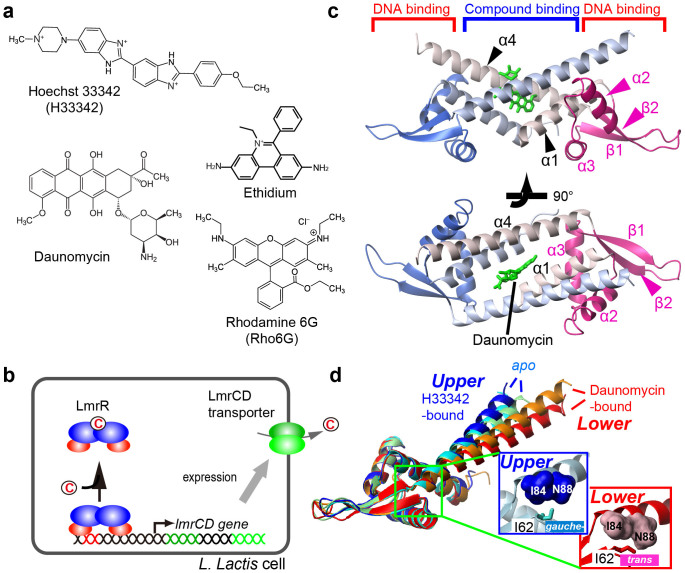
Substrate, regulatory mechanism, and structural features of LmrR. (a), Chemical structures of compounds that bind to LmrR. (b), Schematic representation of gene regulation by LmrR. (c), Ribbon diagrams of LmrR in complex with daunomycin. The compound (green sticks) binds to the pore at the dimeric center. (d), Different orientations of the C-terminal α4 helix relative to the wHTH motif. A superposition of the *apo* (cyan and light green), H33342-bound (blue), and daunomycin-bound (orange and red) LmrR subunit structures is shown. Inset: Close-up view of the hinge region. Ile-62 sidechain is shown as sticks while Ile-84 and Asn-88 are shown in surface representations.

**Figure 2:**
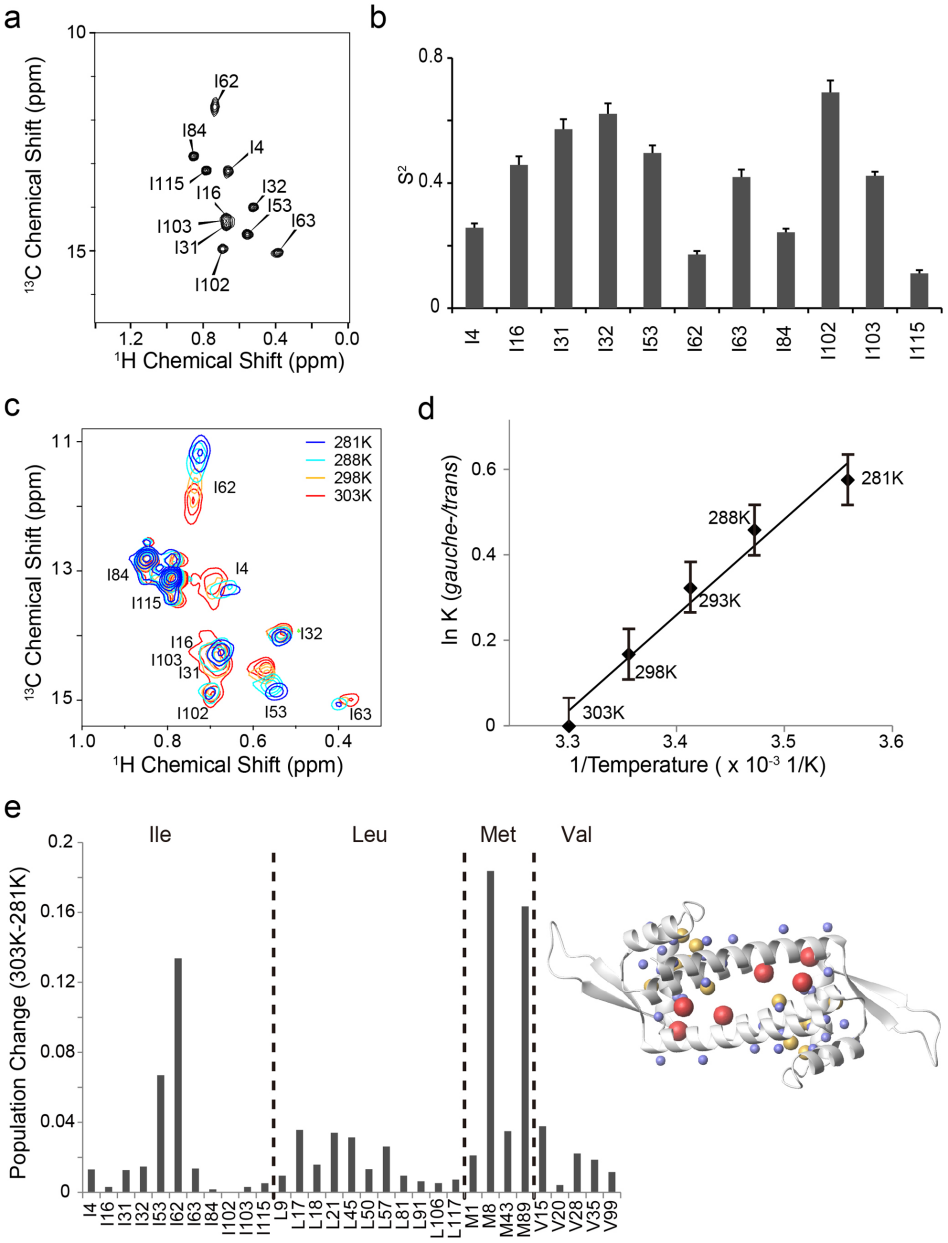
Conformational equilibrium in *apo* LmrR. (a), The Ile δ1 region of the ^1^H-^13^C HMQC spectra of LmrR in the *apo* state. (b), Methyl *S*^*2*^ values of Ile δ1. Error bars correspond to standard deviation (SD) of fitting errors. (c), Temperature dependence of the Ile δ1 resonances of *apo* LmrR. (d), The van 't Hoff plot for the Ile-62 χ2 angle rotational equilibrium in the *apo* state. The error bars correspond to the acquisition resolution of indirect ^13^C dimension. e, Temperature-dependent population change in Ile χ2, Leu χ2, Met χ3, and Val χ1 rotameric states between 281K and 303K. Right: the methyl moieties with more than 10% or 3% rotameric population shift in response to the temperature variation are highlighted in red and yellow, respectively. The methyl moieties that did not show more than 3% rotameric population shift were depicted by small blue spheres.

In the crystal structures of LmrR, a significant variation was observed in the α4 helix orientations, which might reflect its accessible conformational landscape (Fig. 1d). The differences in the α4-helix orientation are reflected in the different rotameric states of the residues in the hinge region that connects the N-terminus of the α4 helix to the wHTH domain. In particular, the χ2 angle of Ile-62 adopts the *gauche*– and *trans* rotameric states when the α4 helix is in the *upper* and *lower* orientations, respectively (Fig. 1d, inset). In the *upper* α4-helix orientations, Ile-62 χ2 is in the *gauche*– rotamer and the δ1 methyl moiety is accommodated in a small pocket, formed by Ile-84 and Asn-88 of the α4 helix. Conversely, in the *lower* orientations, the χ2 angle of Ile-62 is in the *trans* rotamer and the Ile-62 δ1 methyl moiety is rotated away from the α4 helix, due to the steric hindrance. Note that we assumed an exchange between not single, but multiple *upper* and *lower* orientations, based on the X-ray structures of LmrR (Fig. 1d), which demonstrated that *upper* and *lower* α4*-*helix orientations with different tilt angles exist for each rotameric state. Since the Ile χ2 angle is correlated with the ^13^C chemical shift of the δ1 position^14^, the ^13^C chemical shift and line shape of the Ile-62 δ1 resonance would reflect the population of the *upper* and *lower* α4-helix conformations at each state. In the ^1^H–^13^C HMQC spectrum of *apo* LmrR, the Ile-62 δ1 signal is broadened in the ^13^C dimension as compared to the others (Fig. 2a). According to the chemical shift value, the Ile-62 χ2 angle has a 1:1 population between the *gauche*– and *trans* rotamers in *apo* LmrR. Furthermore, the Ile-62 δ1 methyl showed a substantially low order parameter (*S*^*2*^; 0.17 ± 0.01, Fig. 2b), as compared to the others, except Ile-115 in C-terminal disordered region. These results indicated that the Ile-62 χ2 angle in the *apo* state undergoes rotameric exchange, reflecting an equally populated conformational equilibrium of the α4 helix among the *upper* and *lower* orientations.

To further assess the conformational equilibrium observed in the *apo* state, we analyzed the temperature dependence of the methyl resonances from 281 K to 303 K. The ^13^C chemical shift of the Ile-62 δ1 resonance in the *apo* state was significantly shifted to high-field positions at lower temperatures (Fig. 2c). The ^13^C chemical shift difference between 281 K and 303 K was 0.78 ppm. This indicated that the population of the *gauche*– χ2 rotameric state of Ile-62 is increased from 50% to 65% by the temperature reduction from 303 K to 281 K, reflecting the preference for the *upper* α4-helix conformations at low temperature (Fig. 2d). As discussed above, Ile-62 χ2 in the *upper* α4-helix conformation adopted the *gauche–* rotamer and the δ1 methyl moiety of Ile-62 in the *gauche–* rotamer is accommodated in a small pocket, formed by Ile-84 and Asn-88 of the α4 helix, in the X-ray structure of LmrR (Fig. 1d). In agreement with this observation, the *gauche–* rotameric state is calculated to be −4.5 kcal mol^−1^ more enthalpically favorable than the *trans* rotameric state, according to the average slope of the van 't Hoff plot of the Ile-62 χ2 rotameric states (Fig. 2d). It should be noted that the fit of the van 't Hoff's equation appears be non-linear, although the non-linearity seems to be within the experimental errors derived from the acquisition resolution of the indirect ^13^C dimension. The non-linear characteristic might suggest that the heat capacity is changing within the temperature range tested. It should be noted that the dynamics of methyl groups are known to contribute to the heat capacity of a protein, through its local conformational entropy^15^. Fig. 2e shows the temperature-dependent changes in the rotameric populations of the Ile χ2, Leu χ2, Met χ3, and Val χ1 angles. The residues that exhibited substantial temperature-dependent population changes upon temperature reduction were distributed around the α4 helix, further supporting the presence of the conformational equilibrium of the α4 helix in the *apo* state of LmrR.

### LmrR conformation in compound-bound state

**Figure 3:**
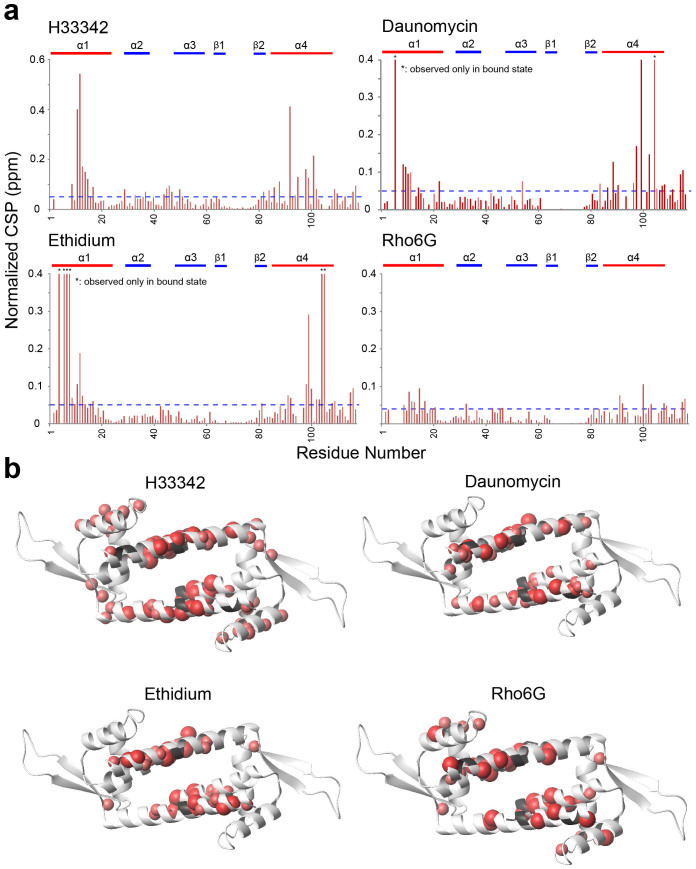
CSPs of mainchain amide resonances of LmrR upon binding to compounds. H33342, daunomycin, ethidium, and Rho6G were added at the indicated concentrations. (a), Plot of the magnitudes of the normalized chemical shift change for each residue. The resonances that were only observable in the compound-bound states are indicated with asterisks. (b), Mapping of significantly perturbed (>0.05 ppm) residues on the ribbon representation of LmrR. Residues that were not observed in each bound state are shown in black.

To characterize the conformational changes associated with compound binding, four known compounds, H33342, daunomycin, ethidium, and Rho6G, which have different sizes and shapes, were titrated to LmrR. SPR analyses indicated that these compounds bind to LmrR with various affinities, with dissociation constants (*K_D_*) values ranging from nM to μM (Supplemental Fig. 2a). The *K_D_* values for H33342 and daunomycin were consistent with those previously reported^13^. The chemical shift perturbations (CSPs) for each amide backbone resonance of LmrR revealed that LmrR engages the compounds with its compound-binding pore (Fig. 3). The amide CSPs induced by compound ligation were mostly converged in the middle of the compound binding site, and thus they directly reflected the proximity of these amides to the compound in the bound states. Therefore, the amide CSPs would vary, depending on the chemical structures of the compounds and their respective binding modes. As expected from the X-ray crystal structures of LmrR, all compounds tested showed 1:1 binding stoichiometry.

**Figure 4:**
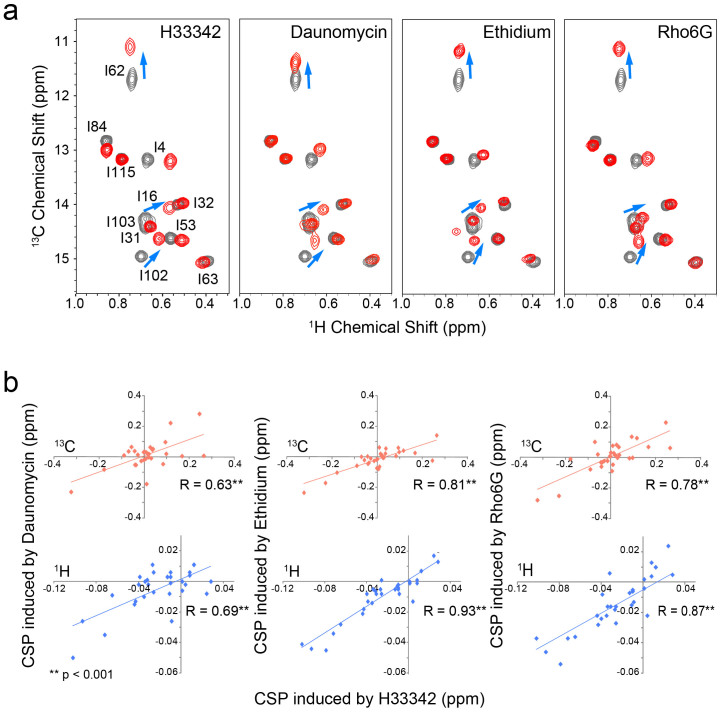
Compound binding induces common conformational changes in LmrR. (a), The Ile δ1 resonaces of LmrR in the *apo* state (black) was overlaid with that of the compound-bound states (red). The resonances with common CSPs are indicated with blue arrows. (b), The pairwise correlation between CSPs induced by daunomycin (left), ethidium (middle), and Rho6G (right) against CSPs induced by H33342, for each resonance that is more than 7 Å away from the compounds. The top and bottom panels are for ^13^C and ^1^H CSPs, respectively. All correlations showed *p*-values less than 0.001.

Interestingly, the binding of different compounds induced similar CSPs to the Ile δ1 signals (Fig. 4a). In addition, the CSPs induced by the binding of H33342 to sites remote from the compound-binding interface, including those from the N- and C-termini of α1 and α4 helixes, correlated well with the CSPs induced by the binding of other compounds (Fig. 4b). Since the methyl groups of Ile δ1 as well as those depicted in Fig. 4b are all far away from the compound binding site, and each chemical shift is a sensitive indicator of its local conformational and dynamical states^14^,^16^,^17^,^18^,^19^,^20^, the correlations between the CSPs from the remote sites shown in Fig. 4b reflect the common global conformational changes that are induced by compound ligation. The observation is a stark contrast to the X-ray structures of LmrR, which showed opposite structural changes upon binding to H33342 and daunomycin. As mentioned above, the orientations of the α4 helix are coupled to the Ile-62 ^13^C chemical shift of the δ1 position^14^. Thus, the substantial high-field shift of the Ile-62 δ1 resonance in the ^13^C dimension, upon compound binding, indicates an elevated proportion of the *upper* orientations in the α4-helix conformational ensemble. The high-field Ile-62 δ1 ^13^C chemical shift changes observed upon compound ligation correspond to 6–12% increase in the *gauche–* rotamer of the Ile-62 χ2 angle, which prefers *upper* α4 helix orientations. It should be noted that the position of the Ile-62 δ1 is always more than 4 Å away from the compound-binding site, and thus its ^13^C chemical shift might not be directly affected by compound binding. It should be also noted that CSPs induced to amide resonances in Fig. 3 would vary, depending on the chemical structures of the compounds and their respective binding modes. In contrast, the Ile and remote methyl CSPs have to be comparable to those induced by the other compounds, as they reflect shared structural change.

### LmrR dynamics in compound bound-state

**Figure 5:**
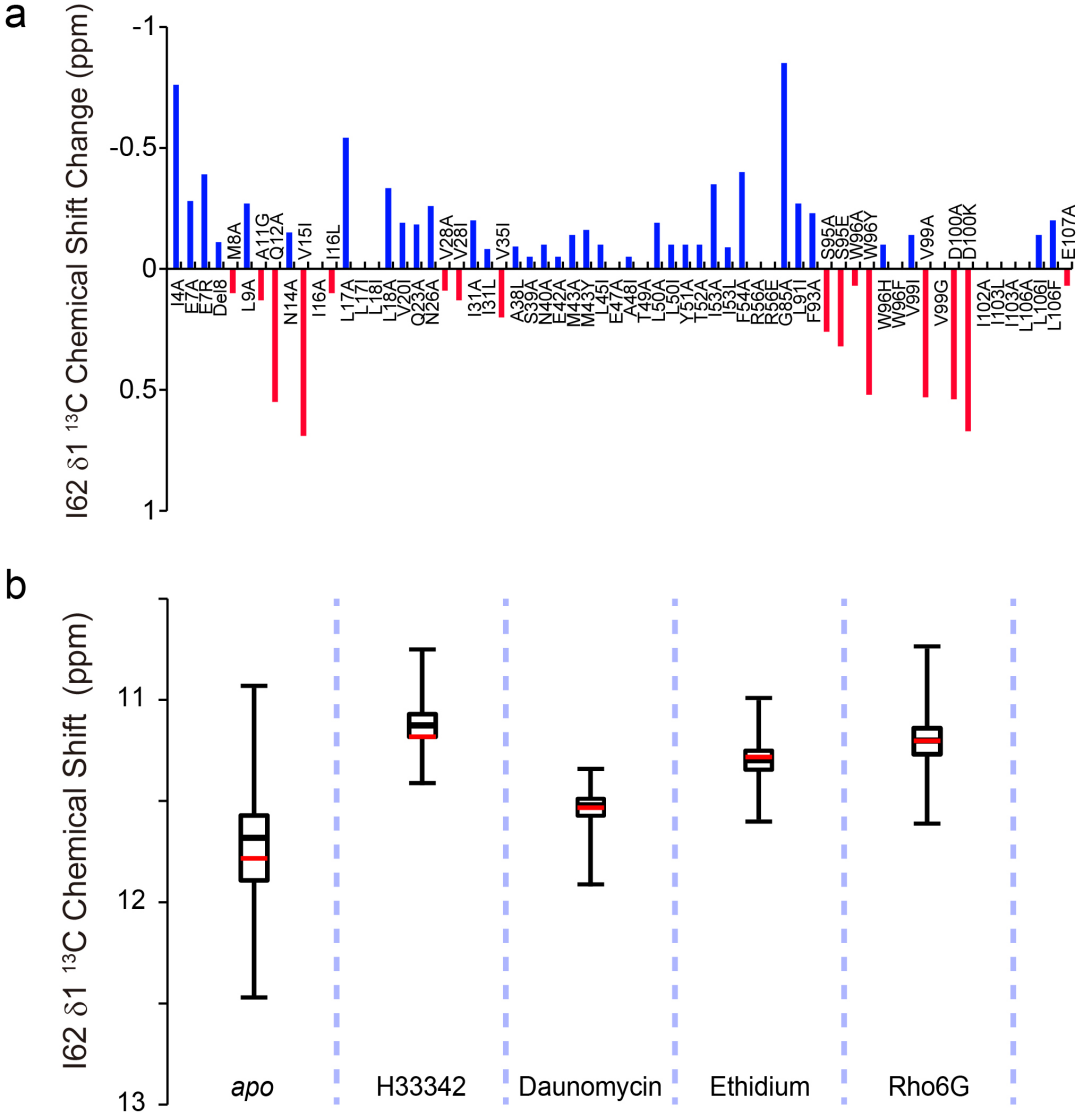
Conformational equilibrium of the α4 helix revealed by allosteric mutations. (a), The ^13^C chemical shift change of the Ile-62 δ1 resonance in each mutant, relative to WT LmrR in the *apo* state. (b), The box and whisker plot of the ^13^C chemical shift of the Ile-62 δ1 resonance in various mutants in the *apo* state as well as in complexes with compounds. The red horizontal line indicates the ^13^C chemical shift of the Ile-62 δ1 resonance of WT LmrR in each state.

To determine the extent of variation of the α4-helix conformations in *apo* LmrR, as well as in the compound-bound states, we prepared 65 mutants of the residues in the α4 helix, and those located at the interface with the α4 helix (Supplemental Fig. 3a). Each mutation introduced a perturbation of the conformational equilibrium of the α4 helix, and the degree of perturbation was monitored by the ^13^C chemical shift of the Ile-62 δ1 resonances (Supplemental Fig. 3b). The ^13^C chemical shift changes in the Ile-62 δ1 resonance, as compared to wild type (WT) LmrR, upon the introduction of each mutation are shown in Fig. 5a. The maximum ^13^C chemical shift change was 0.85 ppm, which is comparable to that induced by temperature changes (Fig. 2c). Thus, the mutants sampled the conformational equilibrium that would reasonably be accessible for WT LmrR.

The ^13^C chemical shifts of the Ile-62 δ1 resonance in the various mutants in the *apo* form, as well as in the compound-bound forms, are shown in Fig. 5b. The Ile-62 δ1 resonance showed a wide distribution of ^13^C chemical shifts, ranging from 11–12.5 ppm, reflecting the shallow energetic potential landscape and the significant variety in the preexisting conformational states in *apo* LmrR. Upon compound binding, the distribution of the ^13^C chemical shifts of the Ile-62 δ1 resonance was shifted to high-field (*i.e.* low-frequency) positions to a different extent for each compound, and the range of the chemical shift variations became smaller. Interestingly, the chemical shift variation observed in the *apo* state includes a substantial part of chemical shift distribution observed in the compound-bound states. This indicated that compound ligation increases the populations of the *upper* α4-helix conformations, among the preexisting conformational ensembles in the *apo* state.

Nevertheless, the substantial temperature dependence revealed by the van't Hoff plot remained in all of the compound-bound states, indicating that the conformational equilibrium of the α4 helix is not fully suppressed in the compound-bound states (Supplemental Fig. 4). Note that the bound-state spectra of LmrR were recorded with at least a 40-fold excess concentration of the compound, as compared to the *K_D_* values, and most of the mutants retained a reasonable binding affinity toward the compounds. Thus, the chemical shift distribution and the temperature dependence are those of the compound-bound states. These results indicated that LmrR recognizes the various structurally unrelated compounds by shifting the equilibrium among the preexisting conformational ensembles in the *apo* state. This notion is further supported by the fact that the mutants that showed extreme populations in the conformational equilibrium exhibited lower affinities toward the compounds (Supplemental Fig. 3c–f).

**Figure 6:**
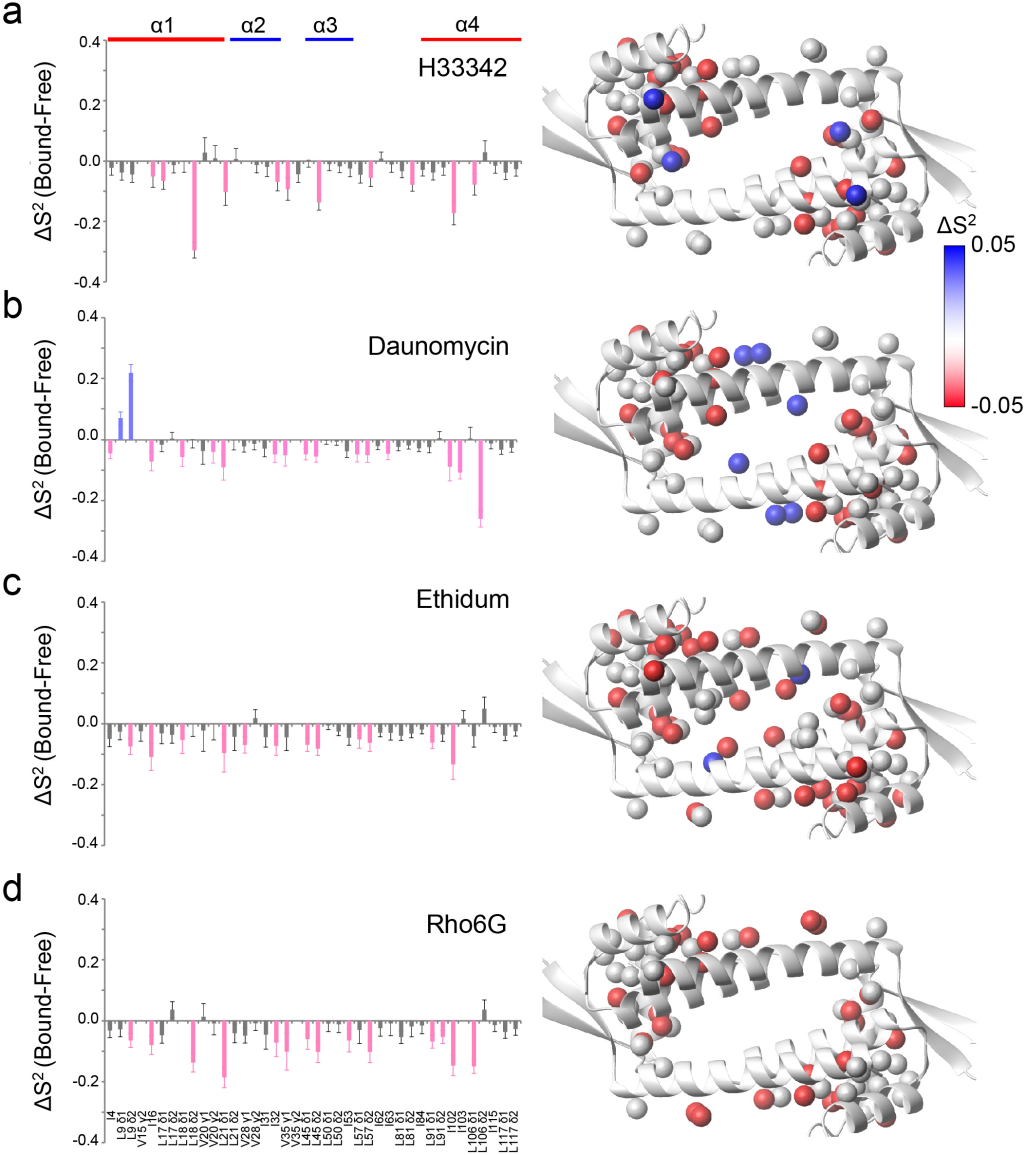
Enhancement of ps–ns motions in allosteric sites upon compound binding. Left panels show Δ*S*^2^ values for each methyl resonance upon binding to (a), H33342, (b), daunomycin, (c), ethidium, and (d), Rho6G. The right panel displays the color-coded mapping of the methyl moieties that showed substantial Δ*S*^2^ values upon binding to each compound. Error bars correspond to SD of fitting errors.

To understand the thermodynamic nature of the LmrR-compound interaction, isothermal titration calorimetry (ITC) analyses were performed. Within the binding free energy of the LmrR-compound interactions, the enthalpic contributions, which reflect the formation of spatially aligned interactions, are small or even unfavorable (Supplemental Fig. 2b and c). While the major entropic term favoring association would be the desolvation from the hydrophobic compound-binding pore of LmrR, changes in the protein conformational entropy would also account for the ligand binding affinity^21^. To determine the contribution of the protein conformational entropy to the LmrR-compound binding, we employed ^1^H spin-based relaxation violated triple-quantum (3Q) coherence transfer NMR spectroscopies, to measure the changes in *S*^2^ (Δ*S*^2^) of the methyl groups upon compound binding^22^,^23^. Since *S*^2^ is a measure of the amplitude of internal dynamics, it is related to the conformational entropy^24^,^25^,^26^,^27^,^28^,^29^,^30^,^31^,^32^,^33^,^34^,^35^. Compound binding to LmrR resulted in a notable increase in the amplitude of ps–ns dynamics throughout the entire protein, except for a few methyl moieties in the compound binding site (Fig. 6). The residues that showed increased ps–ns dynamics are concentrated at the interface between the compound-binding and DNA-binding domains (upper left and lower right corner in the structures shown in Fig. 6), which is allosteric to the compound-binding site and exhibited small amplitude of ps–ns dynamics in the *apo* state (Supplemental Fig. 5). Thus, the compound binding induces the redistribution of the ps–ns dynamics in LmrR, thereby favorably contributing to the binding by increasing the conformational entropy.

### Reciprocal compound/promoter binding

**Figure 7:**
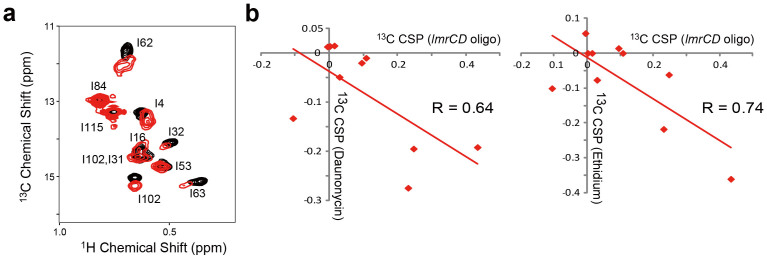
NMR analyses of binding of promoter DNA oligo to LmrR. (a), The Ile δ1 region of the ^1^H-^13^C HMQC spectrum of LmrR in the *apo* state (black) was overlaid with that of the *lmrCD* oligo-bound state (red) (b), The pairwise correlation between CSPs induced by daunomycin (left) and ethidium (right) against CSPs induced by the *lmrCD* oligo for each Ile δ1 resonance.

LmrR shows reciprocal compound- and promoter-DNA-binding properties. To gain structural insights into the reciprocal interactions, we subjected the LmrR–promoter complex to SPR and solution NMR studies. It has been suggested that LmrR interacts with the promoter/operator region of the *lmrCD* gene, including an imperfect inverted repeat (IR) with a PadR consensus. Thus, we used the 33-bp promoter DNA fragment, containing the IR sequence, for the analyses (Supplemental Fig. 6a; hereafter, we refer to this as the *lmrCD* oligo). The SPR analyses indicated that the *lmrCD* oligo showed μM affinity for LmrR (Supplemental Fig. 6b) and was dissociated from LmrR in the presence of the compounds (Supplemental Fig. 6c). Titration of the *lmrCD* oligo to LmrR induced a low-field shift in the Ile-62 δ1 ^13^C chemical shift, which indicated that the conformational equilibrium of the α4 helix was shifted to have higher proportion of the *lower* orientations (Fig. 7a). In addition, the Ile δ1 CSPs induced by the *lmrCD* oligo were anti-correlated with those induced by the compounds (Fig. 7b). Thus, the compounds and the promoter DNA prefer distinct α4-helix conformational ensembles present in LmrR, and provide the structural basis for the reciprocal binding.

## Discussion

**Figure 8:**
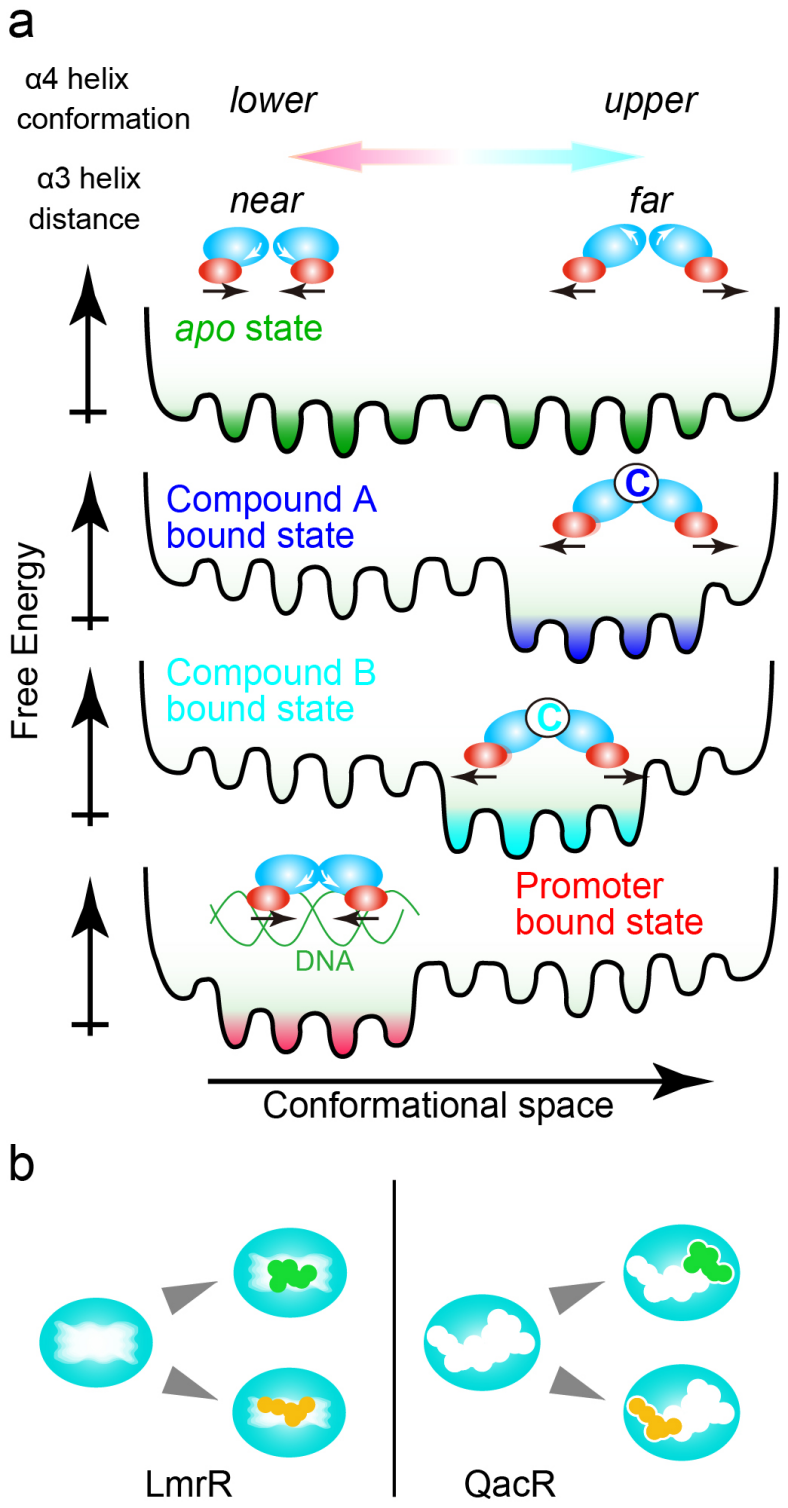
Multidrug recognition and transcriptional regulation through differential shifting of the preexisting conformational equilibrium. (a), In the *apo* state, LmrR exists as a conformational ensemble with multiple α4-helix orientations, in which the *upper* and *lower* conformations are almost equally represented (top). Compound ligation shifts the preexisting conformational equilibrium to various extents, to achieve multidrug recognition (middle). The reciprocal compound/promoter binding by LmrR is achieved by the incompatibility of the conformational ensembles (lower). (b), Comparison of the multidrug recognition by LmrR (left) with that by QacR (right). LmrR binds to multiple compounds using the same site without being locked into a specific conformer. In contrast, QacR possesses multiple specific binding spots in a wide binding pocket to recognize multiple compounds using different sites.

Our structural and dynamical analyses of LmrR in the *apo* state revealed intensive ps–μs dynamics in the compound-binding site. LmrR adopts multiple α4 helix orientations, which are largely classified into the *upper* and *lower* conformations, and each of these conformations should have certain variations in their tilt angles (Fig. 1d). The *upper* and *lower* conformations are associated with *gauche–* and *trans* rotamers in Ile-62 χ2 angle, respectively. As discussed above, the *gauche–* rotamer is enthalpically preferred in the *upper* α4-helix orientations, as it has additional contact between Ile-62 δ1 methyl and the α4 helix. The smallest ^13^C chemical shift value of the Ile-62 δ1 methyl moiety for the H33342 complexes corresponds to 74% of rotameric state in the *gauche–* rotamer (Fig. 5b). Thus, the Ile-62 χ2 angle would, at least in its majority, be in the *gauche-* rotamer when α4-helix is in *upper* orientations. On the other hand, the *lower* orientation forces the Ile-62 χ2 angle to take *trans* rotamer due to steric hindrance. Thus, the average population and rate of exchange between the *upper* and *lower* conformations are reflected in the ^13^C chemical shift and line shape of the Ile-62 δ1 resonance. In the *apo* state, the *upper* and *lower* conformations are almost equally distributed, indicating that those conformers on average are energetically degenerate (Figs. 2d and 8a; top). Theoretically, an energetically degenerate system is the most sensitive to a perturbation that shifts the conformational equilibrium^36^. Thus, *apo* LmrR appears to be optimally suited for sensing and responding to a variety of compounds, by shifting the conformational ensembles to those that differ from *apo* LmrR. There should be a small but appreciable barrier between the *upper* and *lower* α4 helix conformations, judging from a broader ^13^C line width in the Ile-62 δ1 signal (Fig. 2a), which would not be observed if no activation barrier existed between conformations. Considering the facts that ^15^N R_2_ was insensitive in CPMG experiments up to 1 kHz and that the Ile-62 δ1 gives a single line of resonance, the exchange rate between the conformations should be faster than 10^3^ s^−1^ and the apparent activation energy would be smaller than 13 kcal mol^−1^, according to the Arrhenius equation.

The compound binding shifts the conformational ensemble of the α4 helix observed in *apo* LmrR to a higher proportion of the *upper* conformers, but to a different extent for each compound (Figs. 5b and 8a; middle). Thus, there are common structural changes, which have not been identified by the former structural study of LmrR. The multidrug recognition mechanism is somewhat similar to the multi-protein recognition by ubiquitin via a conformational selection mechanism^37^, in which the *bound* conformations of ubiquitin are already present in the free state. However, in stark contrast to the multi-protein recognition by ubiquitin, LmrR possesses only one compound-binding pore, whereas ubiquitin has distinct binding sites on the molecule for different interaction partners. The ps–μs dynamics in the LmrR compound-binding site is not fully suppressed upon compound binding (Fig. 5b and Supplemental Fig. 4), and only limited numbers of the methyl moieties in the compound-binding site showed reduced ps–ns dynamics upon compound binding (Fig. 6). Thus, the loss of protein conformational entropy at the binding interface, which typically accompanies complex formation^21^, was limited in the LmrR-compound interactions.

Interestingly, the compound binding enhanced the ps–ns dynamics in the sites that are allosteric to the compound-binding interface (Fig. 6). The redistribution of the ps–ns dynamics would entropically favor the interactions between LmrR and the compounds. Although the major favorable entropic term associated with the compound binding arises from the desolvation effect, the redistribution of the fast internal dynamics in the allosteric sites would also positively contribute to the compound interaction. From the recently proposed relationship between measured methyl-group *S*^2^ and conformational entropy^38^, −0.5 to −1.5 kcal/mol of entropic energy gain were expected upon compound binding, which increases the binding affinity by 2- to 13-fold. Since LmrR is a sensory molecule that detects various toxic compounds, the high affinities toward the compounds are prerequisite to its function. In this sense, LmrR represents an elaborate system that is compatible with both high-affinity binding and multidrug recognition, via the redistribution of the ps−ns motions upon compound ligation.

In Fig. 8b, we show a schematic representation of the compound recognition by LmrR, compared to that of the TetR-family multidrug transcriptional repressors. The NMR analysis revealed that LmrR recognizes various structurally unrelated compounds by shifting the equilibrium among the conformational ensembles presented in the *apo* form (Fig. 8a and b; left). This recognition mode is quite different from the multidrug recognition by the TetR-family multidrug transcriptional repressors, which utilize multiple specific binding spots in a wide binding pocket to recognize multiple compounds (Fig. 8b; right). The difference is reflected in the thermodynamics of the compound recognition. While the binding of the compounds to LmrR is entropically favored, the compound binding to a TetR-family member, QacR, is enthalpically favored and entropically disfavored, which clearly indicate the specific bond formations that potentially limit the structure of the compound that can be accommodated by QacR^39^. We should emphasize that the multidrug recognition mechanisms of QacR, as with other mechanisms proposed for other families of multidrug transcriptional regulators, rely on the specific protein-drug interactions, and thus the number of the compounds accommodated by the mechanisms is limited. However, the multidrug recognition through a dynamic shift of conformational equilibrium does not rely on prerequisite bond formations, and thus, in principle, is able to accommodate almost unlimited number of compounds with planar aromatic structures that can fit into the single drug binding pore and represents a multidrug binding mechanism, which does not fall into any category proposed thus far.

Lastly, the distinct conformational ensembles preferred by the promoter DNA and the compounds (Figs. 7 and 8a), which we found here, are fundamental to the reciprocal compound/promoter binding by LmrR. This would occur via structural coupling between the α4-helix orientation and the distance between the α3 DNA-recognition helices. The compound binding increases the *upper* conformations of the α4 helix, causing an increase in the average distance between the α3 helices. In contrast, binding to the promoter DNA oligo induces the *lower* conformations of the α4 helix, and thus the shorter distance between the α3 helices would be expected. In the absence of the promoter-bound structure of LmrR, we cannot rule out the possibility of DNA distortion; however, the perturbation of the LmrR conformational ensemble upon binding to the promoter DNA is evident from the promoter-induced CSPs. Ile-62 is located in the hinge region of the α4 helix and it is reasonable that Ile-62 showed a substantial ^13^C CSP. In addition, Ile-57, which is located in the α3 DNA binding helix and interacts directly with the Ile-62 sidechain, also changed its rotameric state from *gauche–* to *trans* when the conformation of the α4 helixes was shifted from *lower* to *upper*. Considering the location of Ile-57 and its correlation to the conformation of the α4 helix, Ile-57, along with Ile-62, would form the structural core to transfer the α4 conformation to α3 DNA binding helix, which serves as a conformation switch that induces LmrR dissociation from the promoter upon compound ligation.

## Methods

### Preparation of LmrR

The DNA fragment (5′-GGCCATGGGCATGGCCGAAATTCCGAAAGAGATGCTTCGTGCTCAGACCAATGTGATTCTGCTGAATGTCCTGAAACAGGGTGATAACTACGTATATGGCATTATCAAACAGGTGAAAGAAGCGAGTAATGGCGAAATGGAACTGAACGAAGCAACTCTCTACACCATCTTCAAACGCCTGGAGAAAGATGGGATCATTAGCAGCTATTGGGGTGATGAATCTCAAGGTGGACGTCGCAAGTACTATCGCTTGACGGAAATTGGCCATGAGAACATGCGGTTAGCCTTTGAATCGTGGTCACGTGTTGACAAGATCATTGAGAATCTGGAAGCGAACAAGAAATCCGAAGCGATCAAACTCGAGTGA-3′) encoding *L. lactis* LmrR, with optimized codon usage for *Escherichia coli* (*E. coli*) protein expression, was synthesized (Operon) and ligated into a pET28b vector (Novagen) at the *Nco I* and *Xho I* restriction sites. The resultant vector expresses LmrR with a C-terminal His6-tag. LmrR mutants were constructed by the QuikChange^TM^ strategy (Agilent Technology). *E. coli* strain BL21(DE3) was transformed with the plasmid. Fresh colonies of the BL21(DE3) harboring the LmrR plasmid were inoculated in 10 mL of LB medium containing 50 μg/mL kanamycin and cultured at 37°C for 4 hrs. The cells were collected by centrifugation and further inoculated into 1 L of kanamycin-M9 medium supplemented with 3 g/L d-glucose and 1 g/L ^15^NH_4_Cl as the sole carbon and nitrogen source, respectively. At an optical density at 600 nm wavelength (OD_600_) of 0.8, 0.6 mM isopropyl-β-d-thiogalactopyranoside (IPTG) was added to induce LmrR expression. The induced culture was further incubated at 27°C for 16 hours. For the expression of deuterated LmrR, D_2_O-based M9 media supplemented with 3 g/L [^2^H_7_/^13^C_6_]-D-glucose or [^2^H_7_]-D-glucose was used, depending on the experimental purposes. For selective ^13^CH_3_-labeling of the Ala, Ile (Ile-δ1), Leu/Val, and Met methyl groups, 50 mg/L of [3-^13^C, 2-^2^H] L-alanine, 100 mg/L of [methyl-^13^C, 3,3-^2^H_2_]-α-ketobutyric acid, 100 mg/L of [3-methyl-^13^C, 3,4,4,4-^2^H_4_]-α-ketoisovaleric acid, and 50 mg/L of [methyl-^13^C] L-methionine were supplemented into the medium 30 min prior to the addition of IPTG. The cells were harvested by centrifugation and frozen at −80°C before purification.

The frozen pellets of LmrR-expressing cells were resuspended in lysis buffer consisting of 50 mM Tris-HCl (pH 8.0), 300 mM NaCl, and 10 mM imidazole, and the cells were disrupted by sonication. LmrR was purified from the supernatant of the cell lysate. The supernatant was applied to a 3 mL COSMOGEL His-Accept column, equilibrated with the buffer, which is same as the lysis buffer. The column was washed with 40 mL of the equilibration buffer, and then LmrR was eluted with 20 ml of elution buffer, containing 50 mM Tris-HCl (pH 7.5), 300 mM NaCl, and 300 mM imidazole. The eluate was concentrated by ultrafiltration, using an Amicon Ultra centrifugal filter unit (molecular weight cutoff 10 K; Millipore), and then passed through a 0.22 μm syringe filter for further purification by size exclusion chromatography (SEC). The sample was applied to a HiLoad Superdex 75 prep grade column (GE Healthcare), equilibrated with 50 mM sodium phosphate buffer (NaPi; pH 6.8) containing 300 mM NaCl. The elution fraction was collected, buffer-exchanged into NMR buffer containing 10 mM NaPi (pH 6.8) and 100 mM NaCl, and stored at −80°C until use.

### NMR experiments

All experiments were performed on either Bruker Avance-600, -700, or -800 MHz spectrometers equipped with room temperature (700 MHz) or cryogenic (600 and 800 MHz) triple resonance probes. All spectra were collected using 10 mM NaPi buffer (pH 6.8) containing 100 mM NaCl in either 90% H_2_O/10% D_2_O or 100% D_2_O, depending on the experiments. The typical concentration of LmrR was 0.1–0.2 mM as a monomer. Unless otherwise noted, the experiments were performed at 298 K. Spectra were processed using TOPSPIN (Bruker Biospin) and analyzed with Sparky. The backbone assignments of LmrR were accomplished using standard TROSY triple-resonance experiments. The two-dimensional ^1^H-^15^N HSQC spectrum of LmrR in the *apo* state showed good overall dispersion (Supplemental Fig. 7A); however, the backbone amide^1^H-^15^N cross-peaks could not be detected for residues 4 and 6–8 in the α1 helix and residues 96, 97, 105, and 106 in the α4 helix. The unobservable residues are all in the compound-binding site, and the intensities of the mainchain resonances were weak in the entire compound-binding site (Supplemental Fig. 7B). Assignments of the Ala, Ile, Leu, Met, and Val methyl resonances of LmrR were performed by combining mutational analysis, J-coupling-based triple resonance experiments ((H)CC(CO)NH, H(CCCO)NH, and HCCH-TOCSY experiments), and an analysis of the inter-methyl ^1^H-^1^H NOE network, based on the crystal structures. For the Leu and Val methyl resonances, stereospecific assignments were achieved by using [2-methyl-^13^C, 4,4,4-^2^H_3_] acetolactate (NMR-Bio) as the precursor. The ^1^H–^13^C HMQC spectra of LmrR with assignments are shown in Supplemental Fig. 7C.

The R_1_ and R_2_ relaxation rates and the (^1^H)–^15^N heteronuclear NOEs of LmrR were measured using published ^1^H-detected 2D NMR pulse sequences^39^. The relaxation delays used were 50, 100, 200, 400, 800, 1200, and 1600 ms for R_1_, and 17, 34, 51, 68, 85, 102, 119, and 136 ms for R_2_ measurements. R_1_ and R_2_ values were determined by exponential fits, and their uncertainties were used as the standard errors of the fitted parameters. The steady-state heteronuclear (^1^H)–^15^N heteronuclear NOEs experiments were performed using a symmetric proton irradiation scheme^40^. The protein saturation period before the ^15^N excitation pulse and the total recycling delay were set to 4.0 s and 8.0 s, respectively, to ensure the maximal development of NOEs before acquisition and to allow complete relaxation of the system. Error estimates were calculated from two separate experiments.

The rotameric equilibria of the Ile χ2, Leu χ2, Met χ3, and Val χ1 angles were deduced from the Ile (δ1), Leu, Met, and Val methyl ^13^C chemical shifts, respectively. The ^13^C chemical shifts of methyl signals are reportedly dependent on the sidechain rotamer, as revealed by theoretical and experimental analyses^14^,^16^,^17^,^18^. The population of the *trans* rotameric state (*p_t_*) for each residue was calculated according to the chemical shift values of the methyl ^13^C signals (*δ_obs_*; ppm) using the equations (1–4) below: 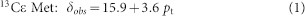
16 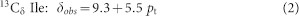
14 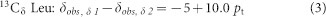
17 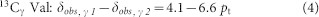
18

If the equation yielded a *p*_t_ value >1 or <0, then the *p*_t_ was fixed to 1 (all *trans*) or 0 (all *gauche*–), respectively.

To measure the order parameters of the methyl groups (*S*^2^_axis_) in LmrR, we employed the ^1^H spin-based relaxation violated coherence transfer NMR spectroscopies^22^,^23^. For this purpose, we used samples in which the methyl groups of Ala, Ile, Leu, Met, and Val residues were ^13^CH_3_-labeled, in an otherwise deuterated background. The intra-methyl ^1^H–^1^H dipolar cross-correlated relaxation rates η were obtained by fitting the ratios of the peak intensities measured in pairs of data sets (*i.e.* forbidden and allowed data) recorded as a function of relaxation time, *T*, to the following equation (5):^22^,^23^. 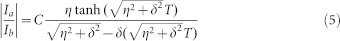
where *T* is the varied delay, *δ* is a parameter related to the density of the external protons around the methyl group, and *I*_a_ and *I*_b_ are the intensities of the forbidden and allowed coherences, respectively, with the delay *T*^22^,^23^. The data sets were recorded using the following sets of relaxation delays *T*: 1, 2, 5, 7, 10, 15, 20, 30, and 40 ms at 298 K. The recovery delay was set to 1.5 s.

*S*^*2*^*_axis_* values were calculated by the determined *η* value using the equation (6) below: 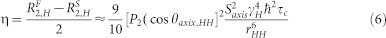
where *τ_c_* is the rotational correlation time of LmrR, *R*^F^_2,H_ and *R*^S^_2,H_ are the relaxation rates of fast and slowly relaxing coherences, respectively, *γ*_H_ is the gyromagnetic ratio of the proton, and *r*_HH_ is the distance between pairs of methyl protons. The rotational correlation time was set to 20 ns, and was determined by TRACT analyses^41^.

The normalized chemical shift perturbation values (*Δδ*) were calculated as follows: 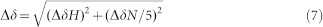


### Surface plasmon resonance (SPR) spectroscopy

All SPR experiments were performed using a BIACORE3000 system (GE Healthcare) at 25°C. LmrR was immobilized to an NTA sensor chip. The binding assay was performed in HBS-P running buffer (10 mM HEPES (pH 7.4), 150 mM NaCl, 50 µM EDTA, 0.005% surfactant P-20) at flow rate of 40 µL/min, using serial dilutions of compounds. Specific binding responses to compounds were obtained by subtracting the response from a flow cell that was not coated with LmrR. For the binding of daunomyin, ethidium, and Rho6G, the equilibrium resonance (R_eq_) from the SPR sensorgrams as function of the compound concentration was plotted and non-linearly fitted to a one-site binding isotherm was used to obtain the *K_D_* values. The binding constant for H33342 was determined by a kinetic analysis assuming 1:1 Langmuir binding. Analyses of the sensorgrams were performed with the BIA-evaluation Software 4.1.

### ITC experiments

Calorimetric titrations of LmrR with compounds were performed with a VP-ITC microcalorimeter (MicroCal) at 25°C, using the same buffer as in the NMR experiments. Protein samples were extensively dialyzed against the buffer before the experiments. The sample cell was filled with a 5–50 µM solution of protein as monomer, and the injection syringe contained 100 μM of H33342, 100 µM of daunomycin, 500 μM of ethidium, or 500 µM of Rho6G. After a preliminary 3 µL injection, 24 subsequent 10 µL injections were performed. Heats were normalized by subtracting those without protein at each titration point to eliminate the effect of dilution. The data were fitted using the sequential binding site model embedded in Origin 7.0 (MicroCal).

## Author Contributions

K.T., Y.T., H.T. and I.S. conceived the project. K.T. and I.M. performed the experiments. K.T., Y.T., H.T. and I.S. wrote the manuscript.

## Supplementary Material

Supplementary Information supplemental figures
