# Supplementary material for: Dynamic multidrug recognition by multidrug transcriptional repressor LmrR
Source: Sci Rep. 2014 Nov 18;4:6922. doi: 10.1038/srep06922 (PMC4235314; doi:10.1038/srep06922)
Supplement: Supplementary Information — supplemental figures [file srep06922-s1.pdf]

Supplemental Information for

Dynamic multidrug recognition by multidrug transcriptional repressor LmrR

Authors: Koh Takeuchi[1], Yuji Tokunaga[1,2,4], Misaki Imai[2], Hideo Takahashi[1,3], and *Ichio Shimada[1,4]

Affiliations:

1. Biomedicinal Information Research Center & Molecular Profiling Research Center for Drug Discovery, National Institute of Advanced Industrial Science and Technology, Aomi 2-3-26, Koto-ku, Tokyo 135-0064, Japan. 2. Research and Development Department, Japan Biological Informatics Consortium, Tokyo, Japan. 3. Graduate School of Medical Life Science, Yokohama City University, Kanagawa 230-0045, Japan 4. Graduate School of Pharmaceutical Sciences, The University of Tokyo, Hongo 7-3-1, Bunkyo-ku, Tokyo 113-0033, Japan.

Supplemental Figures

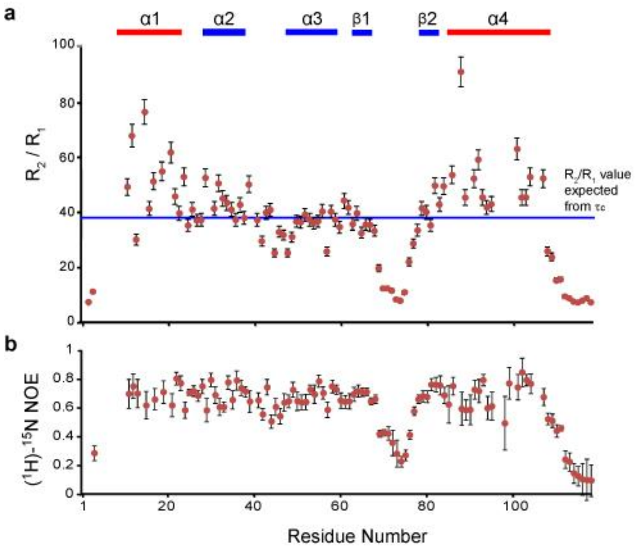

Supplemental Figure 1: The backbone relaxation analysis of LmrR in the *apo* state.

a, The R_2/R_1 ^{15}N relaxation ratios and **b**, the (^1H)–^{15}N NOEs for each residue of LmrR in the *apo* state. In (A), the R_2/R_1 value expected from the global correlation time (τ_c), derived from the TRACT experiment, is shown [42]. The secondary structure elements in LmrR are shown at the top of the panel, with different colors for the compound-binding (red) and DNA-binding (blue) sites. The details of the experimental procedures are provided in the Materials and Methods section.

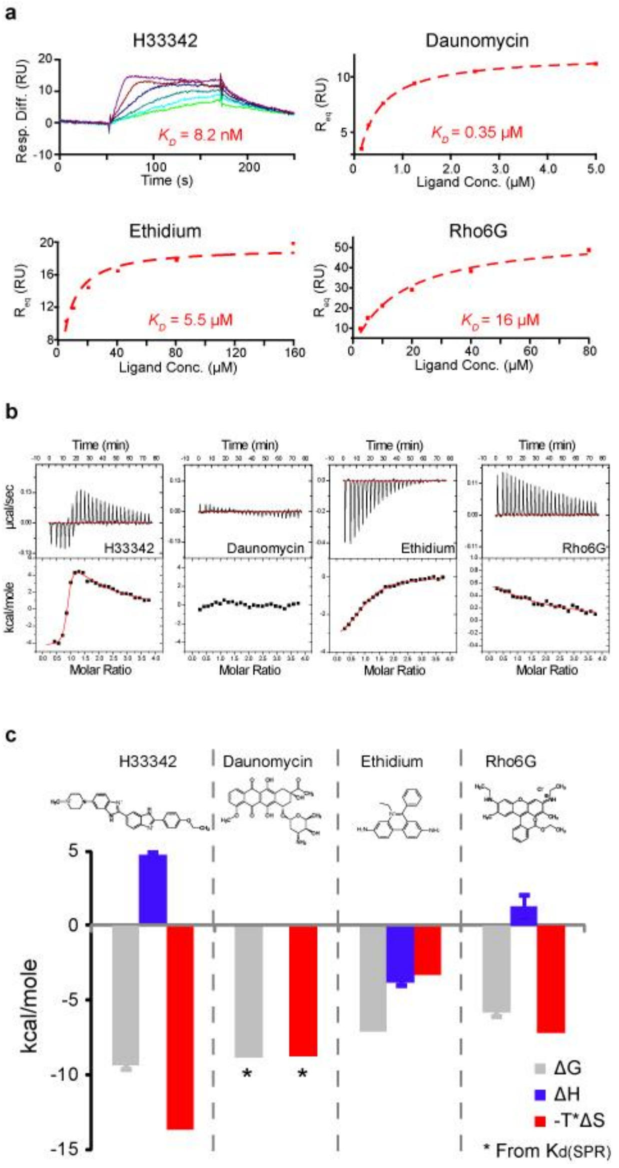

Supplemental Figure 2: SPR and ITC analyses of compound binding to LmrR. a, SPR binding raw signals of H33342 and steady-state response curves of daunomycin, ethidium, and Rho6G to LmrR immobilized on an NTA chip are shown. The dissociation constants were determined by fitting the steady-state response curves assuming a 1:1 interaction for the compounds, except for H33342, which was

determined by global fitting to the SPR raw signals, assuming a 1:1 Langmuir binding isotherm. **b** and **c**, ITC analyses of compound binding to LmrR. **b**, Representative ITC profiles for the calorimetric titration of compounds to LmrR. Representative ITC profiles for the calorimetric titration of compounds to LmrR. Because the binding of daunomycin is purely entropic, the change in enthalpy (or heat generation/absorption) upon binding is inherently too small to be detected by the measurement. **c**, The thermodynamic signatures of the compound-LmrR interactions are shown. The K_D values and the total enthalpy of the binding deduced from the ITC analyses were used to calculate the total free energy change (ΔG) and the total enthalpy change (ΔH), respectively, associated with the binding, except for daunomycin, in which the SPR-derived K_D value was used. The total entropy change (ΔS) was deduced from the difference between ΔG and ΔH.

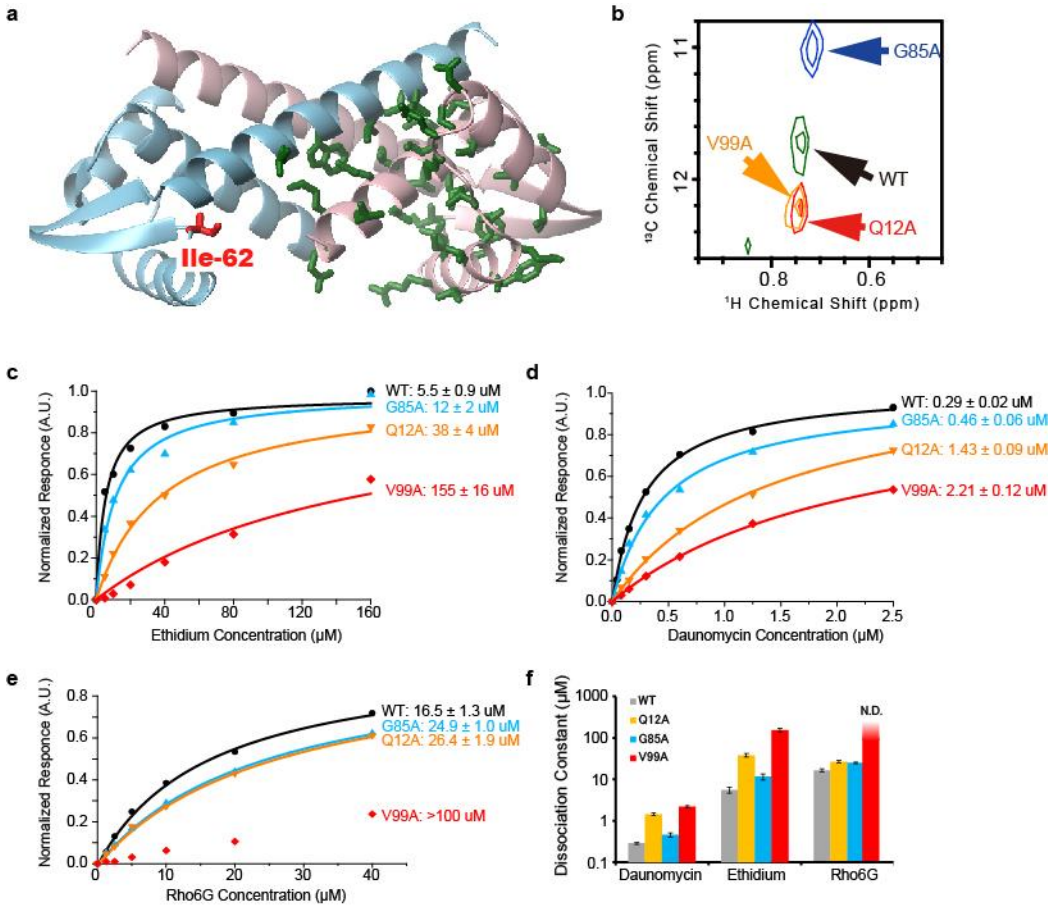

Supplemental Figure 3: The mutations designed to perturb the conformational equilibrium of the α4 helix, and their effects on compound binding. a, All of the residues that were mutated are shown by green stick representations in the apo LmrR structure. The position of lle-62, which monitors the conformational change, is also indicated by a red stick representation. b, The representative chemical shift changes in the Ile-62 δ1 resonances introduced by the mutations. The same mutants that were analyzed in panels c-f are shown. c-f, SPR compound-binding analysis of LmrR mutants that showed extreme *upper* or *lower* populations in the conformational equilibrium of the α4 helix. The conformational equilibrium of the α4 helix was shifted

to the *upper* population in the G85A mutant, while it was shifted to the *lower* population in the Q12A and V99A mutants. Steady-state SPR response curves of c, daunomycin, d, ethidium, and e, Rho6G to LmrR mutants immobilized on an NTA chip. The dissociation constants were determined by fitting the steady-state response curves, assuming 1:1 interactions for the compounds. f, The dissociation constants of each compound to wild type LmrR and the indicated mutants.

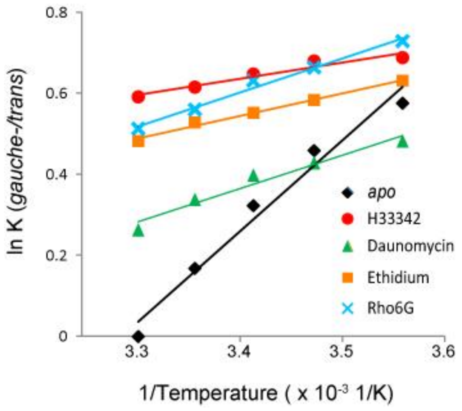

Supplemental Figure 4: The temperature dependence of Ile-62 χ2 angle rotameric equilibria of LmrR in the compound-bound states. The van't Hoff plot for the Ile-62 χ2 angle rotameric equilibrium in the compound-bound states. The Ile-62 δ1 ^{13}C chemical shifts were used to calculate the population of each conformer, assuming the

exchange between the *gauche*- and *trans* rotameric states [14]. The plot for the *apo* state (black) is also shown for comparison.

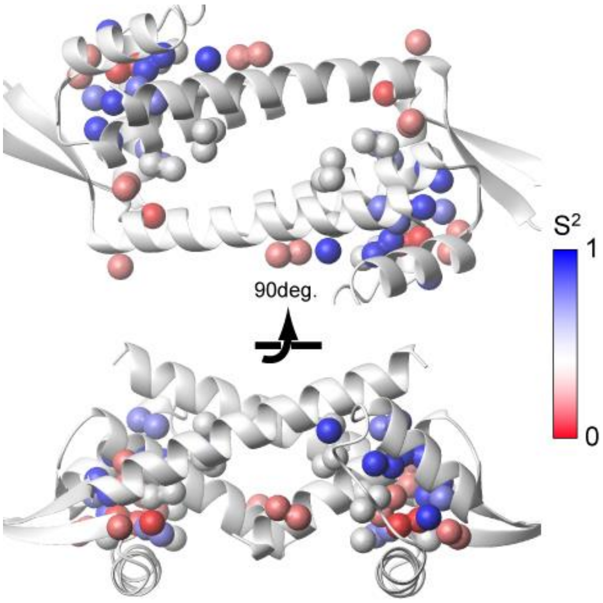

Supplemental Figure 5: The mapping of the ILV methyl S^2 values in the *apo* state.

The methyl S^2 value for each methyl resonance in the *apo* state LmrR is color coded. The ΔS^2 values were calculated from forbidden 3Q experiments [21,22].

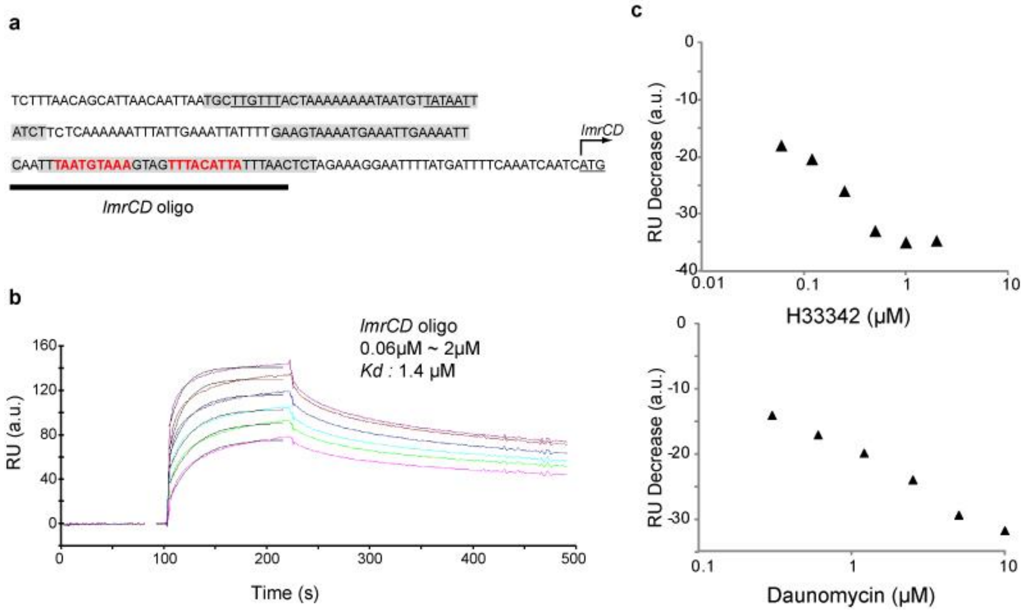

Supplemental Figure 6: SPR analyses of binding of promoter DNA oligo to LmrR.

a,The sequence and the position of the *lmrCD* oligo in the promoter region of the *lmrCD* gene. The position of the *lmrCD* oligo is underlined. The shadows indicate the residues that were identified in a DNA footprinting assay [10]. **b**, Raw SPR binding signals of the *lmrCD* oligo to LmrR immobilized on an NTA chip are shown. The dissociation constants were determined by global fitting to the SPR raw signals, assuming a 1:1 Langmuir binding isotherm. **c**, Dissociation of DNA from LmrR by H33342 (upper) and daunomycin (lower). In the dissociation phase, various concentrations of compounds were injected for 120 sec. Plots of SPR signal reductions by changes with various concentration of compounds are plotted.

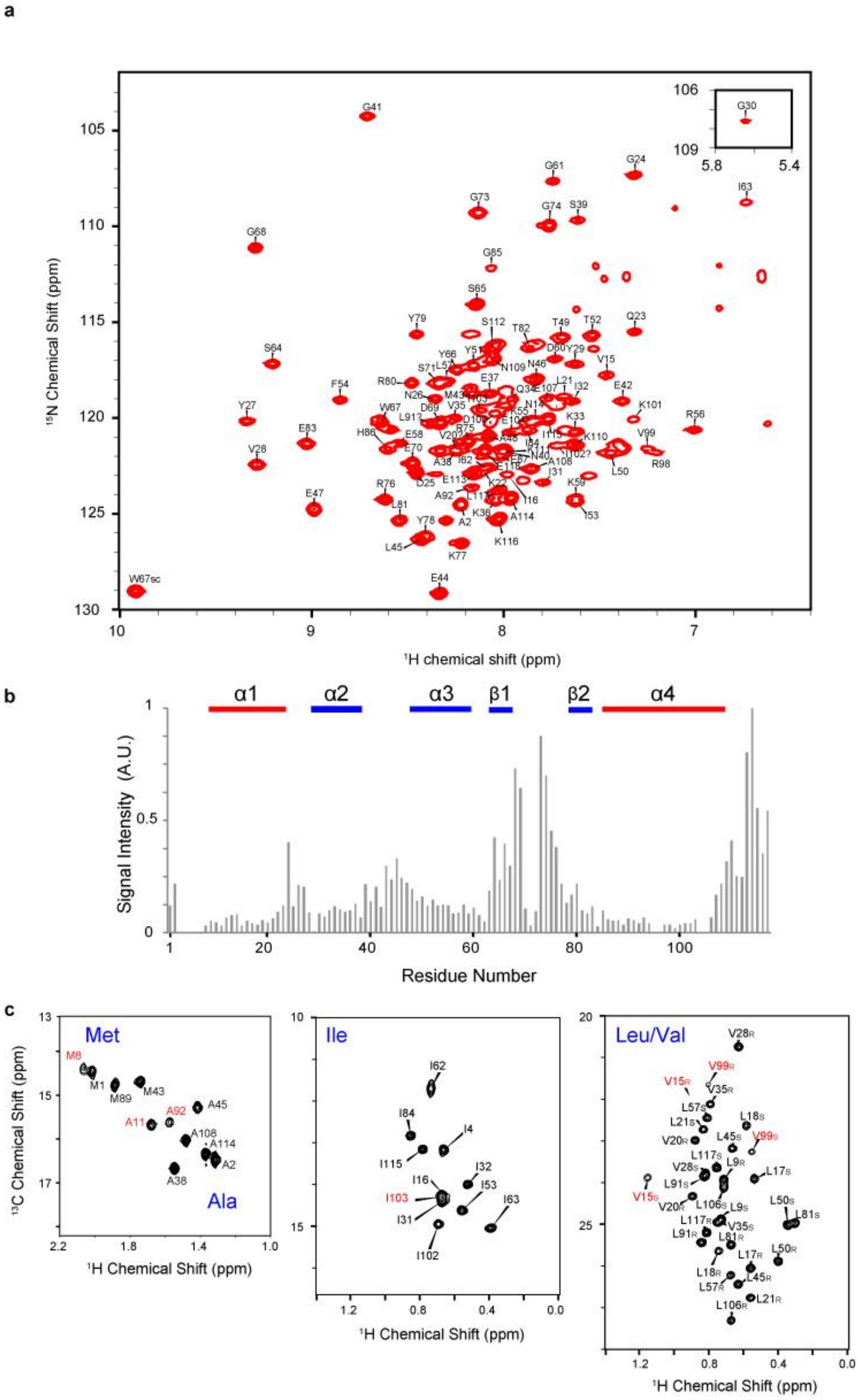

Supplemental Figure 7: NMR analyses of the mainchain and AMILV-methyl resonances of the *apo* form of LmrR. a, Two-dimensional ^{1}H–^{15}N HSQC spectrum of

LmrR in the *apo* state with assignments. **b**, Signal intensity in the HNCO spectrum of LmrR in the *apo* state. The intensity of the $CO_{i-1}N_iH_i$ correlations is shown for each residue. The secondary structure elements in LmrR are shown at the top of the panel, with different colors for the compound-binding (red) and DNA-binding (blue) sites. **c**, Annotated methyl HMQC spectra of LmrR in the *apo* state. Two-dimensional ^{1}H–^{13}C HMQC spectra of [U-^{2}H, AILMV-methyl$^{1}H^{13}C$] LmrR in the *apo* state are indicated with assignments. The resonances from the compound-binding sites are colored red.
